# Nrf2 Is Involved in Maintaining Hepatocyte Identity during Liver Regeneration

**DOI:** 10.1371/journal.pone.0107423

**Published:** 2014-09-15

**Authors:** Yuhong Zou, Joonyong Lee, Shashank Manohar Nambiar, Min Hu, Wenjuan Rui, Qi Bao, Jefferson Y. Chan, Guoli Dai

**Affiliations:** 1 Department of Biology, School of Science, Center for Regenerative Biology and Medicine, Indiana University-Purdue University, Indianapolis, IN, United States of America; 2 Pathology and Laboratory Medicine, School of Medicine, University of California Irvine, Irvine, CA, United States of America; Montana State University, United States of America

## Abstract

Nrf2, a central regulator of the cellular defense against oxidative stress and inflammation, participates in modulating hepatocyte proliferation during liver regeneration. It is not clear, however, whether Nrf2 regulates hepatocyte growth, an important cellular mechanism to regain the lost liver mass after partial hepatectomy (PH). To determine this, various analyses were performed in wild-type and Nrf2-null mice following PH. We found that, at 60 h post-PH, the vast majority of hepatocytes lacking Nrf2 reduced their sizes, activated hepatic progenitor markers (CD133, TWEAK receptor, and trefoil factor family 3), depleted HNF4α protein, and downregulated the expression of a group of genes critical for their functions. Thus, the identity of hepatocytes deficient in Nrf2 was transiently but massively impaired in response to liver mass loss. This event was associated with the coupling of protein depletion of hepatic HNF4α, a master regulator of hepatocyte differentiation, and concomitant inactivation of hepatic Akt1 and p70S6K, critical hepatocyte growth signaling molecules. We conclude that Nrf2 participates in maintaining newly regenerated hepatocytes in a fully differentiated state by ensuring proper regulation of HNF4α, Akt1, and p70S6K during liver regeneration.

## Introduction

Nuclear factor erythroid 2-related factor 2 (Nrf2), a basic leucine zipper region-containing transcription factor, is ubiquitously expressed all over the body, but more predominantly in metabolic organs, including the liver [1]. The various beneficial effects exerted by the activation of Nrf2 in detoxification, antioxidation, and anti-inflammation have attracted numerous investigators to study its regulation, modes of action, target genes, and functions in health and disease, especially over the past decade [2]–[5]. Upon activation by actin-binding Kelch-like ECH-associated protein 1 (Keap1)-dependent and Keap1-independent mechanisms, Nrf2 translocates into the nucleus and regulates the transcription of a network of genes involved in various cellular activities, including redox balance, metabolism, proliferation, and apoptosis. Thereby, Nrf2 is required for maintaining tissue homeostasis. Moreover, several lines of evidence support its important roles in modulating the injury and repair of tissues including the liver [6]–[10]. We are interested in how Nrf2 regulates liver regeneration.

Liver regeneration triggered by partial hepatectomy (PH) is considered to be fundamentally driven by the replication of the remaining mature hepatocytes without the complications of massive cellular injury and inflammation [11], [12]. A recent report highlights the importance of hepatocyte hypertrophy in liver regeneration. Proliferation and hypertrophy of remnant hepatocytes almost equally contribute to the restoration of lost liver mass after 70% liver resection [13]. It has been demonstrated that Nrf2 regulates hepatocyte proliferation by ensuring normal insulin/IGF-1 and Notch1 signaling during liver regeneration [6], [7]. However, it remains unclear whether Nrf2 participates in modulating hepatocyte growth following liver resection. Using the mouse PH model with the presence or absence of Nrf2, we demonstrate here that Nrf2 deficiency results in transient but extensive impairment of hepatocyte growth and identity during liver regeneration.

## Results

### Nrf2 deficiency causes transient but massive reduction in hepatocyte size after PH

**Figure 1:**
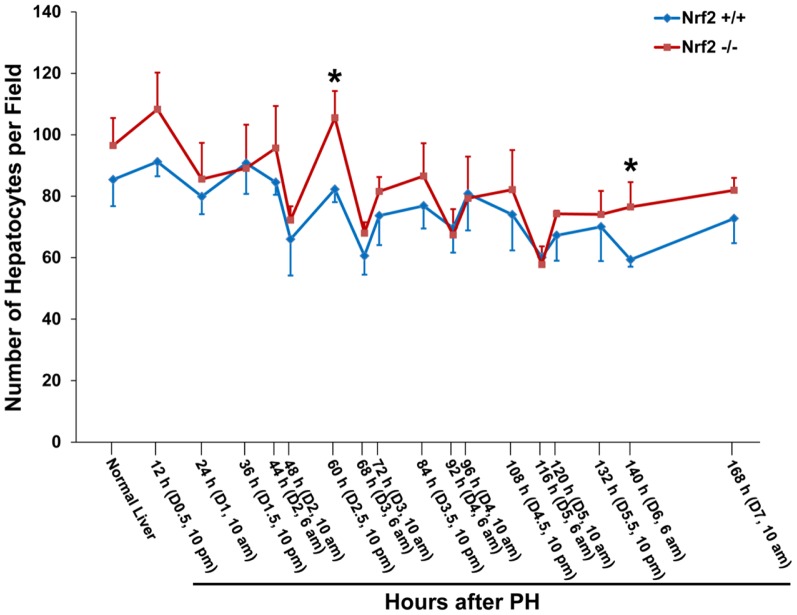
Changes in hepatocyte density after partial hepatectomy (PH) in Nrf2+/+ and Nrf2−/− mice. Livers were collected from the two genotype groups of mice at the indicated time points post-PH. Liver sections were prepared and subjected to hematoxylin and eosin staining. Hepatocytes were counted in five randomly chosen fields per liver section (400x magnification) with Image-Pro Plus software. The results are shown as the means per field ± SD (n = 3 to 5 mice/time point/genotype; *, *p*<0.05 between Nrf2+/+ and Nrf2−/− mice).

**Figure 2:**
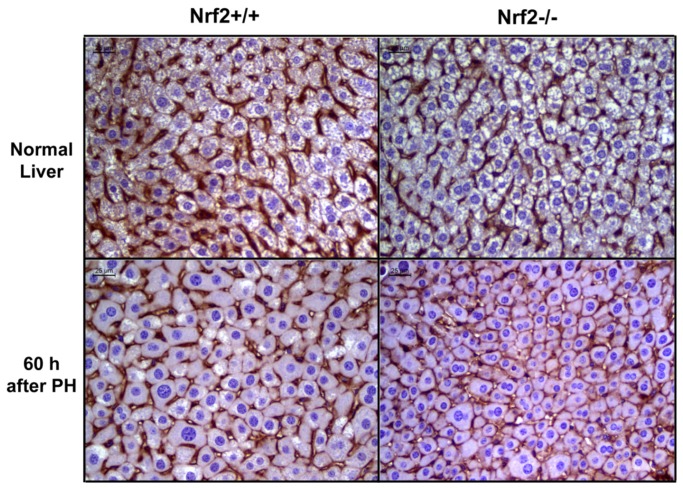
β-catenin immunostaining. Liver sections were prepared from the resting livers and the livers at 60 h after PH in Nrf2+/+ and Nrf2−/− mice and subjected to immunostaining with β-catenin primary antibody. Hepatocyte membrane was stained dark brown.

Hepatocyte hypertrophy constitutes an important but often neglected cellular mechanism in PH-induced liver regeneration [13]–[16]. To determine whether Nrf2 is associated with hepatocyte size regulation, we assessed hepatocyte density during the course of liver regrowth in wild-type and Nrf2-null mice (Fig. 1). Although the average number of hepatocytes per microscope field in normal wild-type mice was lower than that in Nrf2-null mice, statistical significance was not achieved. After PH, the hepatocyte density showed dynamic changes with an overall trend of a decrease in both genotypes of mice. The data indicate that hepatocytes undergo progressive growth (hypertrophy) as liver regeneration advances. Our observation is in line with a report [13]. However, at 60 h and 140 h post-PH, Nrf2-null mice exhibited significantly higher hepatocyte densities than wild-type controls. To clearly visualize hepatocyte sizes, β-catenin immunostaining was performed on tissue sections of the livers collected from both genotypes of mice at 60 h following PH. Obviously, hepatocytes deficient in Nrf2 were smaller than wild-type controls at the time point (Fig. 2). These data suggest that hepatocyte growth was transiently inhibited due to Nrf2 null mutation. This finding prompted us to focus on the temporary event taking place earlier (60 h after PH) to gain insight at the cellular and molecular levels. In addition, we counted binuclear hepatocytes in regenerating livers at 60 h after PH. As a result, there is no significant difference in the percentage of binuclear hepatocytes at this time point between wild-type mice and Nrf2-null mice (wild-types: 13.86±1.2 vs. Nrf2 knockouts: 16.17±3.63, p = 0.23). The data suggest that Nrf2 may not play a role in hepatocyte cytokinesis during liver regeneration.

### Nrf2 deficiency results in transient but extensive activation of liver progenitor markers and depletion of HNF4α in regenerating livers

**Figure 3:**
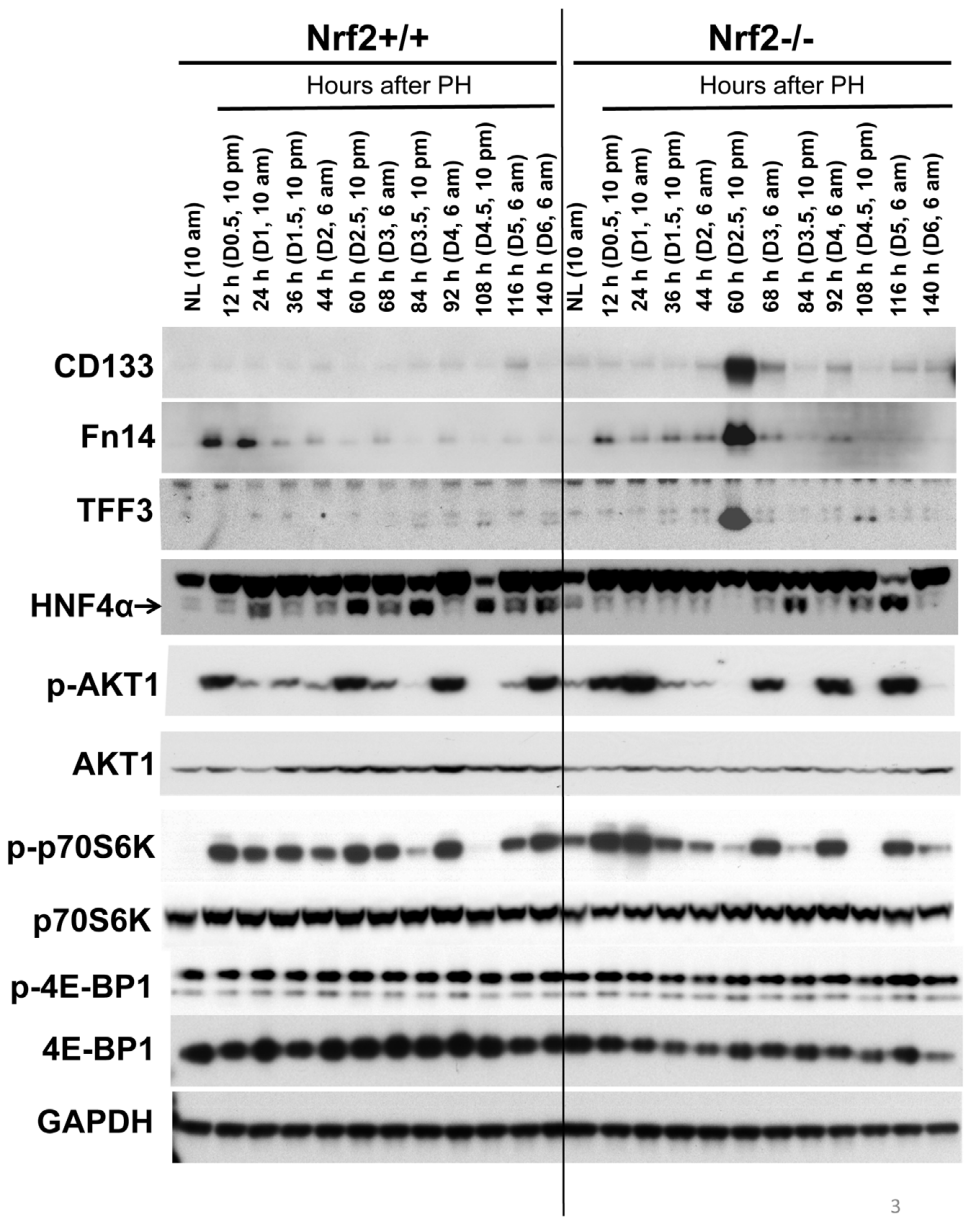
Protein expression of CD133, Fn14, TFF3, HNF4α, p-Akt1 (T308), Akt1, p-p70S6K (T389), p70S6K, p-4E-BP1 (T37/46), and 4E-BP1 in regenerating livers of Nrf2+/+ and Nrf2−/− mice. Livers were collected from normal mice and the mice subjected to partial hepatectomy (PH) at the indicated time points following surgery. Liver lysates prepared from three mice per time point per genotype were pooled with equal amount of protein from each preparation. Western blotting was performed with antibodies against the proteins indicated. Glyceraldehyde 3-phosphate dehydrogenase (GADPH) was used as a loading control. NL, normal liver.

Reduction in size is characteristic of cells undergoing dedifferentiation including hepatocytes [17]. Thus, we hypothesized that hepatocytes lacking Nrf2 may transiently lose their identity during liver regeneration. To test this, we searched for dedifferentiation makers by comparing the gene expression profiles between wild-type and Nrf2-null regenerating livers at the specific time point (60 h after PH). We found that CD133, tumor necrosis factor-like weak inducer of apoptosis (TWEAK) receptor (Fn14), and trefoil factor family 3 (TFF3) were highly upregulated due to the lack of Nrf2. CD133 is widely used as a progenitor cell marker in a number of tissues including the liver [18], [19]. Fn14 has been shown to be expressed in hepatic progenitor cells [20]–[23]. TFF3 is a stable secretory protein predominantly produced by gastrointestinal mucosa and is associated with epithelial restitution [24]–[26]. Very recently, TFF3 was found to be expressed by hepatocellular carcinoma progenitors [27]. We analyzed the protein expression of the three genes throughout the course of liver regeneration in wild-type and Nrf2-null mice. In addition, hepatocyte nuclear factor 4α (HNF4α) was included in the assay because it is essential to the maintenance of hepatocyte differentiation [28]. As shown in Figure 3, the expression of hepatic CD133, Fn14, and TFF3 proteins were drastically activated at 60 h after PH in Nrf2-null, but not wild-type, mice. Wild-type regenerating livers showed marked upregulation in HNF4α protein expression at most timepoints measured. In contrast, the dynamic increases in hepatic HNF4α protein level were prevented prior to 84 h following PH due to Nrf2 absence. Especially, at 60 h after PH, HNF4α protein completely vanished in Nrf2-deficient regenerating livers. Thus, Nrf2 null mutation caused severe dysregulation in HNF4α protein expression post-PH.

**Figure 4:**
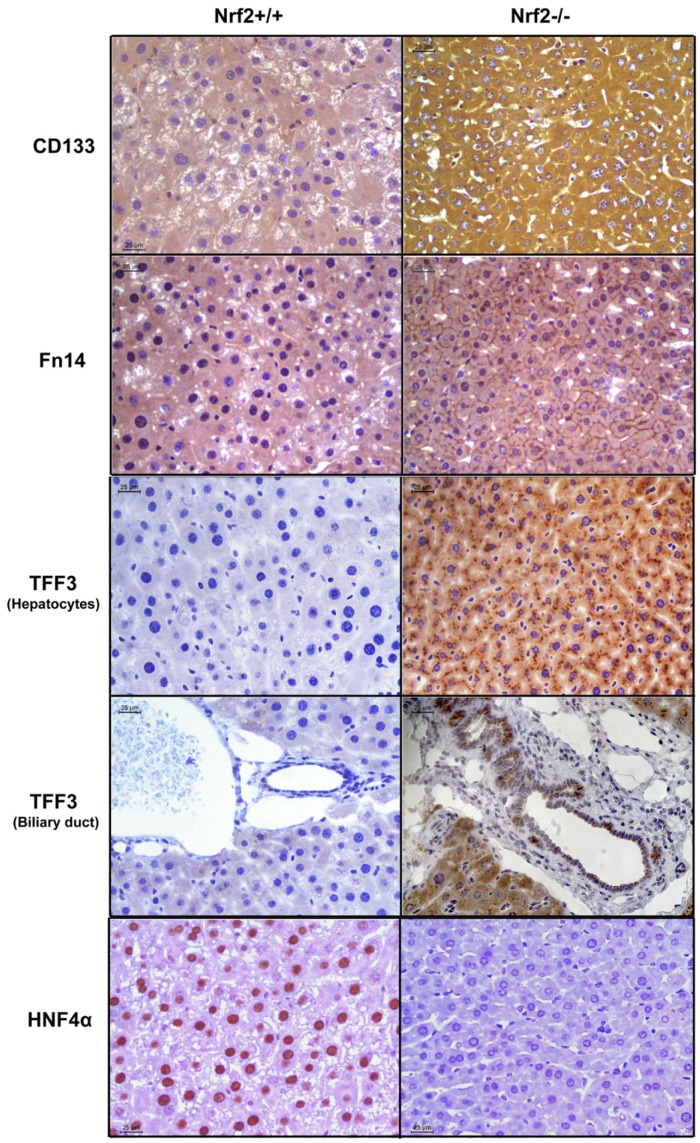
Immunohistochemical analysis of CD133, Fn14, TFF3, and HNF4α in regenerating livers of Nrf2+/+ and Nrf2−/− mice. Liver sections were prepared from the livers isolated at 60 h after PH from three mice per genotype. All liver sections were subjected to immunostaining with primary antibodies against CD133, Fn14, TFF3, or HNF4α. Representative immunohistochemically stained liver sections are shown.

Immunohistochemical analysis revealed the distribution of the four proteins in the liver tissue and hepatocytes (Fig. 4). At 60 h following PH, almost all hepatocytes lacking Nrf2 expressed CD133 evenly in their cytosol, Fn14 on their surface, and TFF3 cytosolically concentrated as aggregates. Simultaneously, HNF4α was abundantly expressed in the nuclei of wild-type hepatocytes, but was not detectable in Nrf2-null hepatocytes. Thus, the vast majority of hepatocytes deficient in Nrf2 exhibited a phenotype of CD133^+^/Fn14^+^/TFF3^+^/HNF4α^−^. In addition, TFF3^+^ biliary epithelial cells were also seen exclusively in Nrf2-null livers at the same time point after PH.

### Nrf2 deficiency leads to downregulation of a group of genes critical for liver function post-PH

**Figure 5:**
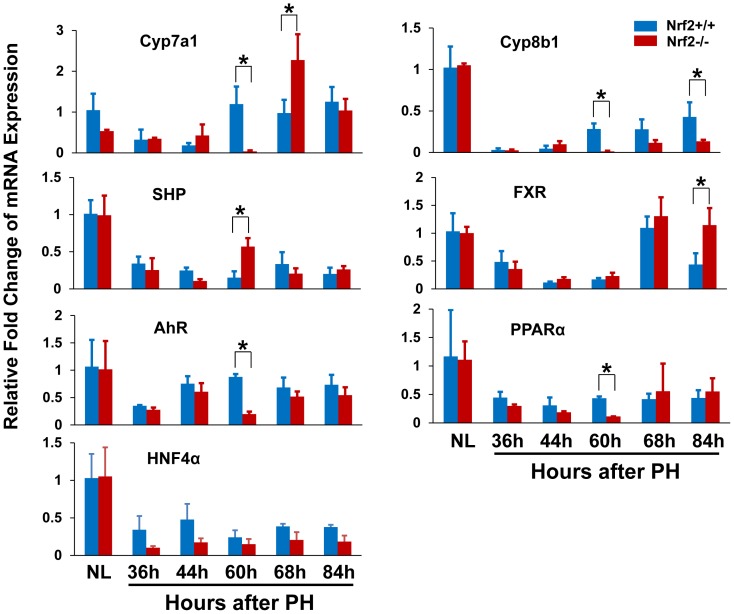
The mRNA expression of a group of genes associated with liver functions in regenerating livers of Nrf2+/+ and Nrf2−/− mice. Total RNA was prepared from the livers at the indicated time points after partial hepatectomy (PH). Hepatic expression levels of the genes indicated were measured by qRT-PCR and are expressed as the means of fold changes relative to the mRNA level in normal livers in Nrf2+/+ mice ± SD (n = 3 mice/time point/genotype; *, *p*<0.05 between Nrf2+/+ and Nrf2−/− mice). NL, normal liver.

Furthermore, we found that a group of genes governing critical hepatocyte functions were markedly downregulated at 60 h following PH due to Nrf2 absence, including bile acid synthesis enzymes Cyp7a1 and Cyp8b1, xenobiotic metabolism-regulating transcription factor Aryl hydrocarbon receptor (AhR), and fat metabolism-regulating nuclear receptor peroxisome proliferator-activated receptor alpha (PPARα) (Fig. 5). At this time point in Nrf2-null regenerating livers, almost diminished expression of Cyp7a1 gene was companied by increased mRNA level of its negative regulator small heterodimer partner (SHP), whereas SHP-positive regulator Farnesoid X receptor (FXR) exhibited PH-dependent downregulation irrelevant to Nrf2. This observation suggests that SHP-mediated prevention of Cyp7a1 expression could be one of the most sensitive responses to the impairment of hepatocyte identity. In addition, we found that hepatic HNF4α mRNA expression was downregulated after PH, irrespective of genotypes. Nrf2 deficiency did not result in significant differences in hepatic HNF4α transcript levels prior to and following PH (Fig. 5). The data are not correlated with PH- and Nrf2-dependent HNF4α protein expression in regenerating liver (Fig. 3). Thus, hepatic HNF4α is regulated at both mRNA and protein levels during liver regeneration and Nrf2-dependent regulation in HNF4α protein expression occurs post transcriptionally.

### Nrf2 deficiency causes dysregulation of Akt and p70S6K activities in regenerating livers

It is known that Akt/mTOR signaling controls the growth and thus sizes of cells, including hepatocytes, via its downstream targets p70S6K and 4E-BP1 [16], [29]. We found that, in contrast to wild-type controls, Nrf2 null mutation led to the inactivation of both Akt1 and p70S6K at 60 h and 140 h after PH (Fig. 3), consistent with the marked reduction in hepatocyte volume at the same time points in Nrf2-null mice (Figures 1 & 2). Following PH, Nrf2 absence resulted in decreases in total hepatic 4E-BP1 protein, but did not severely affect the phosphorylation of the protein, at most time points measured (Fig. 3).

Collectively, we identified a temporal event of massive impairment in hepatocyte identity caused by Nrf2 deficiency during liver regeneration. This event is characterized by the reduction in hepatocyte size, the activation of hepatic progenitor markers, the loss of HNF4α protein, and the downregulation of a subset of genes associated with important hepatocyte functions. Mechanistically, we linked the Nrf2-dependent phenomenon to the depletion of HNF4α protein and the inactivation of Akt1 and p70S6K in regenerating liver.

## Discussion

In this study, we revealed a transient phenomenon of massive damage in hepatocyte identity in a pathological condition (Nrf2 genetic deletion and liver mass loss). This event is most likely indicative of hepatocyte dedifferentiation. We have provided three lines of evidence to support our assumption. Firstly, hepatocytes lacking Nrf2 markedly reduced their sizes at 60 h after PH. Sahin's group reported that mature hepatocytes are able to directly dedifferentiate into liver progenitor cells *in vitro*. Cell condensation or shrinkage is proposed to be one potential unique cellular mechanism underlying mature hepatocyte dedifferentiation [17]. Secondly, simultaneous with the cell volume decrease, hepatocytes deficient in Nrf2 exhibited a dedifferentiation phenotype of CD133^+^/Fn14^+^/TFF3^+^/HNF4α^−^. CD133, Fn14, and TFF3 are known to be coexpressed by hepatic progenitor cells [20], [27]. HNF4α is not only a marker of hepatocyte lineage, but also a master regulator of hepatocyte differentiation [28], [30]. Thirdly, the expression of a subgroup of genes conferring critical liver functions was downregulated and even diminished. It is remarkable that almost all hepatocytes synchronously shrank their sizes, activated hepatic progenitor markers, and reduced the expression of hepatocyte signature genes at this particular stage of live regeneration (60 h after PH) due to the absence of a single molecule (Nrf2). The data reveal an important role for Nrf2 in maintaining newly regenerated hepatocytes in a fully differentiated state during liver repair.

Mammalian cell dedifferentiation *in vivo* has been observed in the pancreas in adult mice [31], [32]. In light of those findings in pancreas, our study strongly suggests that hepatocytes could undergo dedifferentiation in response to a pathological stimulus. A previous study in gene expression profiling performed within 48 hours after PH revealed that replicating hepatocytes reduce the expression of a large set of genes involved in liver metabolic function [33], [34]. This phenomenon is considered as transient transcriptional dedifferentiation of normal mature hepatocytes undergoing proliferation, although the activation of the signature genes characteristic of fetal hepatic cells or HCC was not observed [34]. Here, we found that a subset of hepatic progenitor markers (CD133, Fn14, and TFF3) were activated, following the completion of the first wave of hepatocyte replication (60 h after PH), when Nrf2 was lacking. Our findings implicate that Nrf2 is required to prevent the progression of hepatocyte dedifferentiation during liver regeneration.

Recent reports have highlighted the plasticity exhibited by mature hepatocytes. They are able to dedifferentiate into liver progenitor cells *in vitro* and to transdifferentiate into biliary epithelial cells during chronic liver injury [17], [35]. Notably, Nrf2-null mice exhibit high susceptibility to carcinogenesis induced by several carcinogens or inflammation in multiple organ systems [36]–[39]. Hence, it is very likely that, during the regenerative response to chemical-induced tissue injury, the cells deficient in Nrf2 undergo temporal dedifferentiation, thus promoting tumor formation. Furthermore, TFF3 is associated with the transformation, growth, and migration of cancer cells [40]. Therefore, our findings provide a potential new explanation for the high incidence of carcinogenesis caused by Nrf2 ablation.

It is intriguing how Nrf2 null mutation triggers the loss of mature hepatocyte identity and even hepatocyte dedifferentiation during the process of liver repair. Our study suggests that Nrf2-dependent regulation of the expression of HNF4α protein and the activities of Akt1 and p70S6K may contribute to the event. It has been well established that HNF4α is required for liver development, hepatic specific expression of many genes associated with metabolism, and maintaining hepatocyte differentiation [30]. Among all the time points studied, 60 h post-PH was the only one when the deletion of HNF4α protein and the inactivation of Akt1 and p70S6K occurred simultaneously (Fig. 3). Presumably, at this time point after PH in Nrf2-null regenerating liver, complete loss of HNF4α protein coupled with the inactivation of Akt1 and p70S6K may synergistically exert detrimental effects. These effects trigger hepatocytes to reduce their sizes, activate progenitor markers, and decrease the expression of liver functional genes at the same time. As a result, hepatocytes display severe impairment in their identity and may go dedifferentiation. Notably, at 140 h after PH, Nrf2-null hepatocytes inactivated Akt1 and p70S6K and thereby reduced their sizes compared with wild-type controls (Figures 1 & 3). However, Nrf2-null hepatocytes decreased, but not fully prohibited, the expression of HNF4α protein relative to wild-type hepatocytes at the same time point. This may explain why liver progenitor markers (CD133, Fn14, and TFF3) were not activated at 140 h post-PH in Nrf2-null regenerating livers. Several lines of evidence support a role of insulin/IGF signaling in hepatocyte proliferation and liver growth [41]–[43]. It has been shown that Nrf2 absence results in reduced hepatic insulin/IGF1 signaling at 3 h after PH [6]. Further studies are needed to determine whether the disrupted activities of Akt1 and p70S6K are caused by dysregulated insulin/IGF signaling in Nrf2-null regenerating liver.

A recent report demonstrates that Nrf2 activation exerts negative effects on liver regeneration by delaying proliferation and inducing apoptosis of hepatocytes [8]. However, another recent report shows that Nrf2 displays a beneficial effect by promoting compensatory liver hypertrophy after portal vein ligation [44]. We believe that Nrf2 activity needs to be tightly controlled during liver regeneration, because both overly activated and deficient Nrf2 activity impairs hepatic regenerative response.

In summary, we identified a Nrf2-depedent phenomenon of hepatocyte identity impairment during liver repair. We presume that, as liver regeneration proceeds, Nrf2 presence enables HNF4α to keep newly regenerated hepatocytes in a differentiated state and allows Akt1 and p70S6K to exert appropriate growth signaling, thus maintaining hepatocyte identity in regenerating livers.

## Materials and Methods

### Animal care and use

The mice were housed in plastic cages at 22±1°C on a 12-hour light/12-hour dark cycle with lights on from 6:00 am to 6:00 pm. Standard rodent chow and water were provided *ad libitum* throughout the entire feeding period. Wild-type and Nrf2-deficient male mice (3 months old) of a C57BL6/129SV mixed background were used for the study [1]. Standard two-thirds liver resections were performed following the procedure described previously [45], [46]. The gall bladders were kept intact. The surgery was performed between 10:00 am and 12:00 pm to minimize the potential variability in the progression of liver regeneration associated with the surgical time and the circadian clock [47]. All of the animal experiments were conducted in accordance with the National Institutes of Health Guide for the Care and Use of Laboratory Animals. Protocols for the care and use of animals were approved by the Indiana University-Purdue University Indianapolis Animal Care and Use Committee.

### Immunohistochemistry

Formalin-fixed and paraffin-embedded liver sections were subjected to a standard procedure of immunohistochemistry. Primary antibodies against β-catenin (BD Biosciences, San Jose, CA), CD133 (orb18124, Biorbyt, San Francisco, CA), Fn14 (ab109365, Abcam, Cambridge, MA), HNF4α (SC-6556, Santa Cruz Biotechnology, Santa Cruz, CA), and TFF3 (PAB19439, Abnova, Walnut, CA) were used.

### Western blot analysis

Liver homogenates (10 or 30 µg) were separated by polyacrylamide gel electrophoresis under reducing conditions. Proteins from the gels were electrophoretically transferred to polyvinylidene difluoride membranes. Primary antibodies used include p-p70S6K (T389) (#9234), p70S6K (#2708), p-4E-BP1 (T37/46) (#2855), and 4E-BP1 (#9644) (Cell Signaling Technology, Danvers, MA); Fn14 (ab109365, Abcam, Cambridge, MA); TFF3 (SC-18273), HNF4α (SC-6556), and glyceraldehyde 3-phosphate dehydrogenase (GADPH) (SC-25778) (Santa Cruz Biotechnology, Santa Cruz, CA); CD133 (PAB12663, Abnova, Walnut, CA); and Akt1 (#1081-1) and p-Akt1 (T308) (#2214-1) (Epitomics). Immune complexes were detected using an enhanced chemiluminescence system (Pierce, Rockford, IL).

### Quantitative real-time polymerase chain reaction (qRT-PCR)

Total RNA was isolated from frozen liver tissue using TRIzol reagent according to the manufacturer's protocol (Invitrogen, Carlsbad, CA, USA). cDNAs were synthesized from the total RNA (1 µg) of each sample using a Verso cDNA Kit (Thermo Scientific, Rockford), diluted four times with water, and subjected to qRT-PCR to quantify mRNA levels. TaqMan Universal PCR Master Mix and the primers and TaqMan MGB probes of mouse Cyp7a1 (mm00484150_m1), Cyp8b1 (Mm00501637_s1), SHP (Mm00442278_m1), FXR (Mm00436425_m1), AhR (Mm00478932_m1), PPARα (Mm00440939_m1), HNF4α (Mm01247712_m1) and albumin (Mm00802090_m1) were purchased from Applied Biosystems (Foster City, CA). The amplification reactions were carried out with the ABI Prism 7900 sequence detection system (Applied Biosystems, Foster City, CA) with initial hold steps (50°C for 2 min followed by 95°C for 10 min) and 40 cycles of a 2-step PCR (92°C for 15 seconds and 60°C for 1 min). The comparative CT method was used for the relative quantification of the amount of mRNA in each sample normalized to the albumin transcript levels.

### Hepatocyte density measurement

Formalin-fixed and paraffin-embedded liver sections were stained with hematoxylin and eosin. Hepatocytes were counted with Image-Pro Plus software (Media Cybernetics, MD, USA) in five randomly chosen microscope fields at 400x magnification for each sample.

### Statistical analysis

Data are shown as the means ± standard deviation (SD). Statistical analysis was performed using a one-way analysis of variance. Comparisons of means were determined by post-hoc analysis. Significant differences were defined when *P*<0.05.
